# Growth and size control during development

**DOI:** 10.1098/rsob.170190

**Published:** 2017-11-15

**Authors:** Jannik Vollmer, Fernando Casares, Dagmar Iber

**Affiliations:** 1 D-BSSE, ETH Zürich, Mattenstrasse 26, 4058 Basel, Switzerland; 2 Swiss Institute of Bioinformatics (SIB), Mattenstrasse 26, 4058 Basel, Switzerland; 3 CABD, CSIC-Universidad Pablo de Olavide-JA, 41013 Seville, Spain

**Keywords:** growth control, growth termination, *Drosophila*, mathematical models

## Abstract

The size and shape of organs are characteristic for each species. Even when organisms develop to different sizes due to varying environmental conditions, such as nutrition, organ size follows species-specific rules of proportionality to the rest of the body, a phenomenon referred to as allometry. Therefore, for a given environment, organs stop growth at a predictable size set by the species's genotype. How do organs stop growth? How can related species give rise to organs of strikingly different size? No definitive answer has been given to date. One of the major models for the studies of growth termination is the vinegar fly *Drosophila melanogaster.* Therefore, this review will focus mostly on work carried out in *Drosophila* to try to tease apart potential mechanisms and identify routes for further investigation*.* One general rule, found across the animal kingdom, is that the rate of growth declines with developmental time. Therefore, answers to the problem of growth termination should explain this seemingly universal fact. In addition, growth termination is intimately related to the problems of robustness (i.e. precision) and plasticity in organ size, symmetric and asymmetric organ development, and of how the ‘target’ size depends on extrinsic, environmental factors.

## 1. Introduction

### 1.1. Intrinsic versus extrinsic growth control

**Figure 1.**
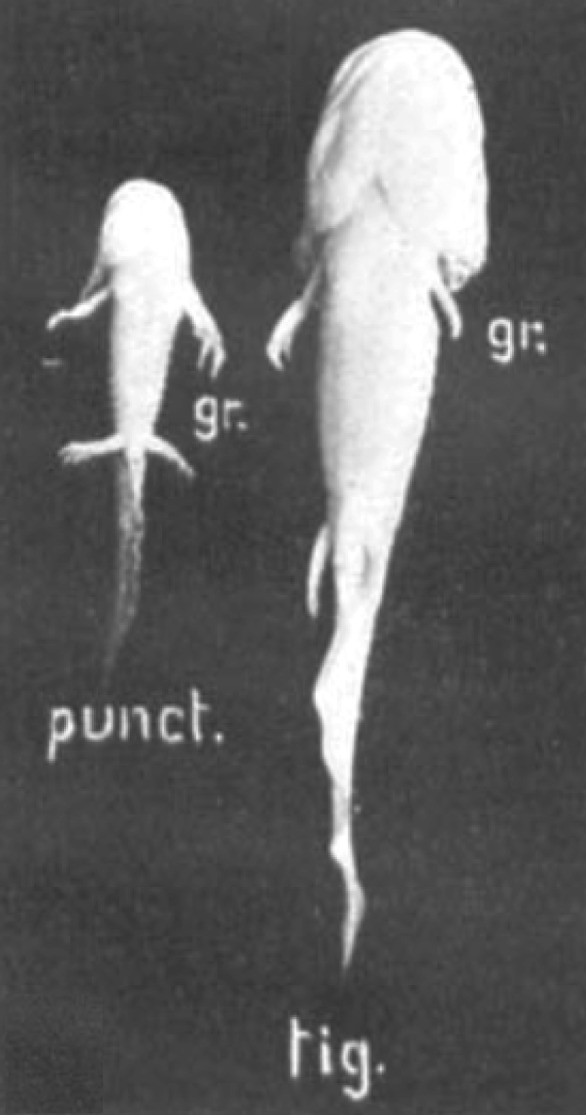
Intrinsic organ size control. Grafting experiments demonstrate intrinsic growth control. Limbs were transplanted between embryos of *A. punctatum* and *A. tigrinum*. The grafted limbs (gr.) grow with comparable kinetics and to a similar size as their non-grafted controls. The picture shows animals 40 days after operation. Reproduced with permission from Twitty & Schwind [8] (copyright © 1931 John Wiley and Sons).

At the beginning of the twentieth century, Harrison [1] introduced transplant experiments to evaluate the relative contributions of organ-intrinsic (autonomous) and organ-extrinsic (non-autonomous) growth control. Initial studies of heteroplastically transplanted organs showed ambiguous outcomes regarding the importance of organ-extrinsic and organ-intrinsic control of growth [1–7]. Twitty & Schwind [8] introduced a strategy of maximal feeding that led to the maximal possible growth rate in donor, recipient and transplant. In this way, Twitty & Schwind were able to separate intrinsic and extrinsic factors (e.g. nutrition) in a controlled and stereotyped way. They grafted eyes and limbs at the tail-bud stage between two differently sized salamander species: *Ambystoma punctatum* (now known as *Ambystoma maculatum* or spotted salamander) and *A. tigrinum* (or tiger salamander). In the populations used, *A. tigrinum* grows to approximately 1.8 times the size of *A. punctatum* [1]. The same size ratio was also observed for the larval stages studied, even though the larvae were initially of the same size [8]. When organs were grafted between embryos of these two species at the tail-bud stage, Twitty & Schwind found that the growth of the graft was comparable with the growth of the control organ that remained on the donor salamander. Thus, the eyes or limbs grew with comparable kinetics and to approximately the same size as they would have done had they not been transplanted (figure 1) [8]. This finding was even more astonishing, considering that the limbs first appear at different developmental time points in these species and that the species in general differ greatly in their growth kinetics. A similar finding was also reported for transplantation experiments between *A. punctatum* and the axolotl [8], and in heterochronic transplantations of wing buds between chicken embryos [9,10], indicating its general validity.

Intriguingly, organs employ different mechanisms to adjust their size, and the relative contribution of organ-intrinsic and organ-extrinsic factors can vary, depending on the organ of interest, even within a single species. Thus, Metcalf [11,12] found that multiple fetal thymus glands transplanted into a developing mouse each grow to their normal size, while multiple fetal spleens grow to the mass of one adult spleen. Each spleen itself stays smaller such that the total mass is equivalent to one normal spleen. These experiments suggest that the growth of the thymus glands is regulated by organ-autonomous or organ-intrinsic factors while the growth of the spleens seems to be controlled by some negative feedback mechanism that monitors the external environment of the developing organ (i.e. by organ-extrinsic factors).

That multiple smaller spleens can make up for one normal-sized spleen suggests that the smaller spleens develop the same functionality, yet on a smaller developmental domain. Developmental processes indeed often scale with changes in developmental domains and time scales. Examples include mutations in insulin-related genes that result in substantially smaller (twofold), but perfectly proportioned flies [13], as well as manipulations in frog embryos that result in smaller, but perfectly patterned tadpoles [14].

In summary, organ growth is controlled by both the organ-intrinsic and the organ-extrinsic mechanisms and the relative contributions of the control mechanisms differ between organs. In this review, we will focus on the intrinsic mechanisms that ensure organ growth termination. Readers interested in organ-extrinsic mechanisms and pattern scaling should refer to reviews and primary papers in the field [15–24].

### 1.2. *Drosophila* imaginal discs as model systems

Given the complexity of the growth control mechanisms, significant insights have been gained from studying a simple model system, the *Drosophila* imaginal discs.

**Figure 2.**
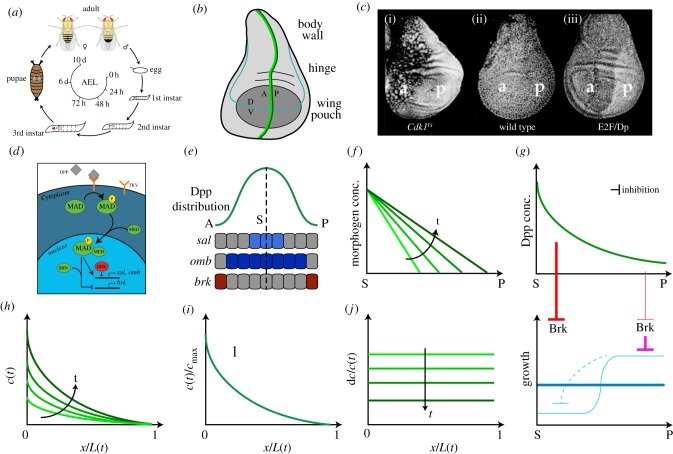
The *Drosophila* wing imaginal disc as a model system for growth control. (*a*) Life cycle of *Drosophila*. The adult fly deposits the fertilized eggs, in which embryogenesis is happening, into the food. Approximately 1 day after egg laying (AEL), embryogenesis is completed and the larvae hatch from the eggs. The larval stage takes approximately 4 days in total and includes two moults, from first to second instar at roughly 48 h AEL and from second to third instar at roughly 72 h AEL [25,26]. Before undergoing pupation, the larvae stop feeding (approx. 5 days AEL) and enter the wandering stage at which they search for a dry place. During pupation, metamorphosis takes place and the adult body structures are assembled from the imaginal discs. (*b*) Cartoon of a third instar *Drosophila* wing disc. The wing blade develops from the so-called wing pouch (dark grey). Other parts of the disc form the connection between wing and body (hinge) or parts of the thorax. The dorsal–ventral (DV, light blue) and anterior–posterior (AP, dark green) boundaries, as well as the expression zone of Dpp (light green), are indicated. (*c*) Inhibiting (i) or enhancing (iii) the cell cycle alters the size of the cells, but not the overall size of the wing disc (wild-type disc in ii). Constitutive overproduction of dE2F increased expression of the S- and M-phase initiators Cyclin E and String (Cdc25), thereby accelerating cell proliferation. The pictures were adapted from Neufeld *et al.* [27]. Reprinted with permission from Elsevier. (*d*) Simplified illustration of the Dpp signalling pathway. Mad gets phosphorylated upon binding of Dpp to its receptor Tkv. Together with Med and Schnurri (Shn), Mad upregulates expression of the downstream targets *sal* and *omb* and inhibits the expression of the transcriptional repressor *brk*. In the absence of Dpp signalling, *brk* is expressed and Brk inhibits the expression of *sal* and *omb*. (*e*) Distribution of Dpp and expression domains of the target genes *sal*, *omb* and *brk* in the *Drosophila* wing disc. *dpp* is expressed just anterior to the AP compartment boundary (*b*) (S, source) and forms a bidirectional gradient (green). Dpp induces the expression of *sal* (light blue) and *omb* (dark blue) and downregulates the expression of *brk* (*d*), limiting its expression to the lateral sides (red)*.* The expression zone of *omb* is wider than the one of *sal* due to their different sensitivities to Dpp. (*f*) Growth control by morphogens: the gradient slope model as originally proposed by Day & Lawrence [28]. According to this model, cells sense the slope of the (linear) Dpp gradient and proliferate proportionally to the slope. As time progresses, the slope decreases and cell proliferation slows down accordingly (light to dark green). (*g*) Growth control by morphogens: the growth equalization model proposed by Schwank *et al.* [29]. According to this model, growth in the wing disc pouch is inherently inhomogeneous (light blue). In the absence of Dpp, lateral cells have a growth advantage and over-proliferate (light blue). This over-proliferation leads to an inhibition of proliferation in the medial part of the tissue (dashed, light blue). Dpp leads to an equalization of the growth, such that the resulting growth rate is homogeneous throughout the tissue (dark blue). Dpp restricts the expression of Brk to the lateral parts of the tissue (red, thicker lines indicate a stronger inhibition). Brk prevents over-proliferation (pink), thereby also releasing the inhibition of proliferation in the medial part of the pouch. (*h–j*) Growth control by morphogens: the temporal dynamics model proposed by Wartlick *et al.* [30]. According to this model, cells divide every time they sense a certain relative increase in Dpp signalling levels. (*h*) The amplitude and the length of the Dpp gradient both expand on the growing wing disc domain *L*(*t*) over developmental time (light to dark green). (*i*) All gradient profiles collapse on a single curve, if normalized with the maximal Dpp concentration (*c*_max_) and domain length *L*(*t*) (i.e. the gradients scale). (*j*) For an exponential, scaling gradient with linearly increasing amplitude, the relative change in the concentration d*c*/*c*(*t*) is equal within the entire domain and declines with developmental time (light to dark green). Thus, it takes progressively longer to reach the relative increase needed to trigger cell proliferation and growth will terminate.

The life cycle of *Drosophila* consists of embryogenesis, which happens in the fertilized egg, three larval stages, instar one to three (which are separated by moults), pupation, during which metamorphosis takes place, and finally, the adult stage as fully developed fly (figure 2*a*). Imaginal discs grow mainly during the larval stages as the primordia for most external body structures of the adult fly, for example the wings and the eyes (figure 2*b*). At the beginning of the first instar, the primordium of the wing imaginal disc consists of approximately 30 cells, but a complete disc can be generated even from as little as four to six founder cells [31]. The cells then normally undergo 9 to 11 rounds of cell divisions [31,32]. As the cell numbers increase, the disc also changes its appearance from a ‘flattened sac’ to a much more buckled epithelium with the disc proper on the one side and the peripodial membrane on the opposing side. While the disc proper consists of cells of columnar shape, the peripodial membrane consists of squamous cells. One part of the disc proper, the so-called wing pouch, develops into the wing blade and has attracted most of the research (figure 2*b*). Other parts of the disc proper form the hinge, the connection between wing and body, and parts of the thorax. Another approximately two rounds of divisions are happening during the pupal stage [33]. Estimates for the cell number at metamorphosis range from 30 000 [32] to 50 000 cells [34]. The wing emerges by eversion (i.e. via turning inside-out through the larval wall to the free space in the pupal case) [35]. As the cells of the wing do not divide or grow after eversion [28], the final size of the wing disc at this point determines the final size of the adult wing.

There are several observations to suggest that also in imaginal discs, growth control is based, to a large extent, on organ-intrinsic mechanisms [36]. Somewhat comparable with the transplantation of limbs, imaginal wing or eye discs grow to their normal size even if under completely different external conditions. Thus, when imaginal discs were dissected from developing larvae and transplanted into the abdomen of adult flies, they developed, even if at a lower developmental speed, to resemble the size and shape of normal discs [34,37]. The growth stopped independently of the age of the host, but solely based on the age and size of the transplants [37]. Similarly, regenerative growth of fragments of discs transplanted to abdomens of adult flies can result in normal-sized discs [38,39].

So, how do imaginal discs sense their size and adjust their growth rate accordingly? Several mechanisms and models have been proposed to explain growth termination in an organ-intrinsic manner. Here, we provide an overview, and discuss experimental evidence in favour and against them, focusing mainly on evidence obtained from research on *Drosophila* discs.

## 2. Models for controlling growth termination

### 2.1. Growth control by limiting the number of cell division events?

The counting of cell division events would present a straightforward mechanism for autonomous growth termination. Independent of the speed of development, such a mechanism would result in the same final cell number and, if cells maintained the same size, in the same final size of the wing disc. Several groups tested this possibility by manipulating the cell cycle and concluded that tissue size control is not achieved via counting cell divisions [27,40]. Thus, completely blocking cell divisions in the pupal stage, and to some extent even in larval stages, did not alter the final size of the wing disc [40]. Similarly, manipulation of the cell cycle length by either overexpressing or blocking expression of the transcriptional regulator dE2F or its corepressor RBF altered cell numbers over a four- to fivefold range, but did not affect final wing size (figure 2*c*) [27]. Intriguingly, manipulations of cell size in a single compartment still resulted in a correct final anterior-/posterior-compartment size ratio, as judged by visual inspection rather than exact quantification [27].

It should be noted that this conclusion applies beyond invertebrates. Thus, cells of the eastern newt (*Triturus viridescens*) are normally diploid (i.e. they contain two homologous sets of chromosomes) [41]. With increase or decrease in the number of chromosome sets (haploid = 1, triploid = 3, tetraploid = 4 or even up to pentaploid = 5), which occurs naturally but can also be enforced through experimental techniques, the cells increase and decrease in size, respectively. This cell size change is compensated on the body and organ level by the number of cells, such that the embryos are of about the same size [41–43]. Similar findings were also observed in mice [44]. Different organs appear to employ different mechanisms to achieve such size compensation. Thus, in the case of glandular organs, size was either compensated by the increase of the individual tubules while keeping their numbers constant or by increasing the number of tubules while keeping their size constant [41]. These observations show that different mechanisms appear to ensure patterning robustness in case of alterations in size as well as the existence of a range of mechanisms to correct sizes by compensating growth.

### 2.2. Growth termination by limiting developmental time?

Limiting the total developmental time would constitute another straightforward mechanism. Martín & Morata exploited the effect of the *Minute* mutation (which reduces the protein synthesis rate) to study the impact of additional developmental time on final wing disc size by generating slow-growing *M*/*+* larvae with normally growing Minute^+^ (*M^+^*) wing discs [45]. The homozygous *Minute* mutation is lethal for *Drosophila*, but heterozygous *Minute* mutants (*M*/*+*) develop, even if more slowly than wild-type controls due to a decreased mitotic rate [46,47]. They estimated that, using this system, the discs are provided with 20 h of additional growth time. With an average cell cycle length of approximately 10 h, this could have resulted in an additional two rounds of cell divisions or, assuming a constant cell size, a fourfold increase in disc size. Somewhat surprisingly, there was no significant size difference of the wing disc at prepupal stage or of the adult wings, indicating a disc-intrinsic mechanism for growth termination [45]. There was also no apoptosis of any ‘excessive’ cells. By generating mosaic discs, in which either the anterior (A) or posterior (P) compartment was *M^+^*, they could also show that the initial difference in compartment sizes, due to the different growth rates, disappears by the end of development, suggesting that the mechanism of growth arrest works independently in the A- and P-compartment [45]. In conclusion, discs stop their growth upon reaching the appropriate size, even if provided with additional developmental time [45].

Taken together, these results further support a disc-autonomous mechanism that terminates growth upon reaching the final size. This growth-terminating mechanism does not work based on a simple cell-counting mechanism or on the developmental time available.

### 2.3. Is growth termination controlled by morphogens?

Morphogens have been mainly studied as regulators of patterning, but have a well-documented impact also on final organ size. The BMP2-encoding gene *decapentaplegic* (*dpp*) has attracted the most attention as a growth-controlling morphogen, because it is expressed in all 15 imaginal discs (hence its name Decapentaplegic), and ectopic expression of *dpp* or of an activated form of its receptor Thickveins (Tkv) leads to overgrowth [48–53]. By contrast, reduction of its activity reduces wing size drastically and clones of cells mutant for the receptor or downstream genes fail to grow [48,54–59].

Secreted from a stripe of cells just anterior to the AP border of the wing disc (figure 2*b*), Dpp forms a bidirectional gradient (figure 2*e*) [60–63]. Binding of Dpp to Tkv leads to the phosphorylation of the transcription factor Mothers against dpp (Mad, pMad), which regulates downstream gene expression (figure 2*d*) [64–68]. Thus, pMad forms a complex with Medea (Med) and induces the expression of *spalt* (*sal*) and *daughter of dpp* (*dad*), and downregulates the expression of the transcriptional repressor *brinker* (*brk*) (figure 2*d*,*e*) [50,51,68–73]. Brk negatively regulates the expression of *optomotor-blind* (*omb*), and pMad thus also has an indirect positive impact on *omb* expression (figure 2*d*,*e*) [50].

There are two key questions that need to be answered when considering Dpp as a regulator of growth/size: (i) How can the graded distribution of Dpp lead to the uniform proliferation observed in the wing disc [33]? (ii) How can growth termination at the right size be explained? Several models have been developed to address either or both questions. To distinguish between those, the models can be separated into ‘instructive’ and ‘permissive’ models [74]. Instructive models assign the role of growth regulation to Dpp and thus address both questions at the same time. Permissive models, on the other hand, explain only how Dpp can lead to a uniform growth rate [74], and thus depend on other mechanisms for growth termination. The most important models of both types are discussed in the following sections.

### 2.4. The threshold model

According to the threshold model, cells require a Dpp concentration that is higher than a certain threshold to divide. As the disc expands, cells at the lateral edges of the disc will eventually fall below this Dpp threshold and stop dividing. According to the threshold model, the disc terminates growth once the most lateral cells stop dividing. The threshold model thus postulates a binary all-or-nothing response to the Dpp levels. This is, however, inconsistent with the observation that the growth of lateral cell clones with constitutively active Dpp is faster than that of wild-type clones [58]: if there was a binary all-or-nothing response to Dpp, these clones should proliferate at the same speed as the wild-type clones.

### 2.5. The gradient slope model

The gradient slope model, originally proposed by Day & Lawrence [28], states that cells sense the slope of the Dpp gradient. In a refinement of the model, it was suggested that only medial cells require the sensing of the slope while lateral cells respond to absolute Dpp levels, basically following a threshold model [53]. Growth ultimately terminates because the slope or the relative spatial difference becomes progressively smaller with the scaled expansion of the gradient (figure 2*f*). In both cases, it is assumed that the Dpp gradient is linear, such that the slope is constant within the domain, thereby explaining the uniform growth pattern. Quantitative measurements, however, demonstrate that the Dpp gradient is of exponential shape [30,75]. To explain uniform growth with an exponential gradient, it was proposed that cells sense the relative spatial difference of the Dpp concentration along their surface [74]. If growth was indeed controlled by the slope of the Dpp gradient, then uniform Dpp-dependent signalling should lead to a proliferation arrest. Expressing of an activated form of the Dpp receptor Tkv (Tkv^QD^) in the expression domain of the Spalt (*sal*) gene, using the *sal-Gal4* driver, however, did not alter growth, thus contradicting the proposed mechanism [29].

### 2.6. The growth equalization model

The growth equalization model provides only a solution to the question of uniform growth. It does not tackle the question of growth termination and thus belongs to the category of ‘permissive’ growth models. Based on their findings that a gradient of Dpp signalling is not required for normal wing disc development, Schwank *et al.* [29] proposed the growth equalization model. According to their model, Dpp is only required to equilibrate the intrinsically non-homogeneous proliferation through the disc (figure 2*g*). Thus, they propose that in the absence of Dpp, growth is much stronger in the lateral parts of the disc compared with the medial part as lateral cells inhibit the proliferation of the medial parts through an unknown mechanism (figure 2*g*, light blue). The suggested role of Dpp is then to equilibrate these differences by constraining the expression of the repressor Brk to the lateral parts of the discs, thereby reducing proliferation in the lateral parts and releasing the inhibition of proliferation in the medial part [29,74] (figure 2*g*, dark blue). Rather than promoting proliferation directly, in this model, Dpp acts through the repression of Brk. The growth equalization model has recently been supported by the finding that in the absence of Dpp dispersal, lateral cells continue to divide at rates comparable with wild type while patterning and growth in the medial part are lost [76].

### 2.7. The temporal dynamics model

In contrast to the growth equalization model, the temporal dynamics model is an ‘instructive’ model. This model was motivated by the finding that the length of the exponential Dpp gradient scales with the length of the growing wing disc and that its amplitude increases about linearly with developmental time (figure 2*h*,*i*) [30]. As a result of these gradient dynamics, each cell in the tissue experiences the same relative change in the Dpp concentration over time (figure 2*j*). Based on this observation, it was postulated that every time a cell senses a certain relative increase in Dpp signalling, it divides [30,77]. Since all cells experience the same relative increase in the Dpp concentration (figure 2*j*), such a mechanism can explain uniform proliferation. Moreover, because the amplitude in the Dpp gradient increases about linearly with time (figure 2*h*), it takes increasingly longer to reach this relative increase (figure 2*j*). Intriguingly, the predicted rate of slow-down matched the observed decline in the growth rate. To be able to sense a relative increase in the Dpp concentration, the cells would have to adapt to each relative increase of the Dpp signal in a consistent manner. The authors tested their model by conditionally expressing Tkv^QD^, the constitutively active form of the Dpp receptor, in cell clones, thereby exogenously modulating the relative increase sensed by the cells. Indeed, the observed proliferation rates were increased as predicted by the temporal dynamics model [30].

The model is, however, not consistent with other experimental observations. First of all, discs that are mutant for both *dpp* and *brk* overgrow [29], suggesting a permissive role for Dpp, as proposed in the growth equalization model. Moreover, clones which lack *mad* and *brk* grow comparable to wild-type clones, despite the genetic abrogation of the transduction of the Dpp signal [78]. Finally, the findings that Dpp is not required for lateral wing disc growth [76] and during the latter half of larval development [79] also argue against a temporal dynamics model. While the latter finding has meanwhile already been challenged, recent work indeed indicates that low, uniform levels of Dpp are sufficient for promoting normal, homogeneous wing disc growth, while the higher signalling levels within the gradient are necessary for patterning only [80–82].

The observed scaling of the Dpp gradient with the growing wing disc can be explained with the diffusion-based dispersal of the Dpp gradient [83]. Consistent with the actual measurements [30], the scaling is then not perfect, and the imperfect nature of the scaling ensures that the gradient can define expression boundaries for *sal* and *dad* based on a constant concentration threshold, even though the gradient amplitude increases continuously [84]. At the same time, imperfect scaling also means that the relative change in the Dpp concentration differs throughout the wing disc domain, such that the temporal dynamics model would lead to neither uniform growth nor growth termination.

### 2.8. Growth termination by cell differentiation

**Figure 3.**
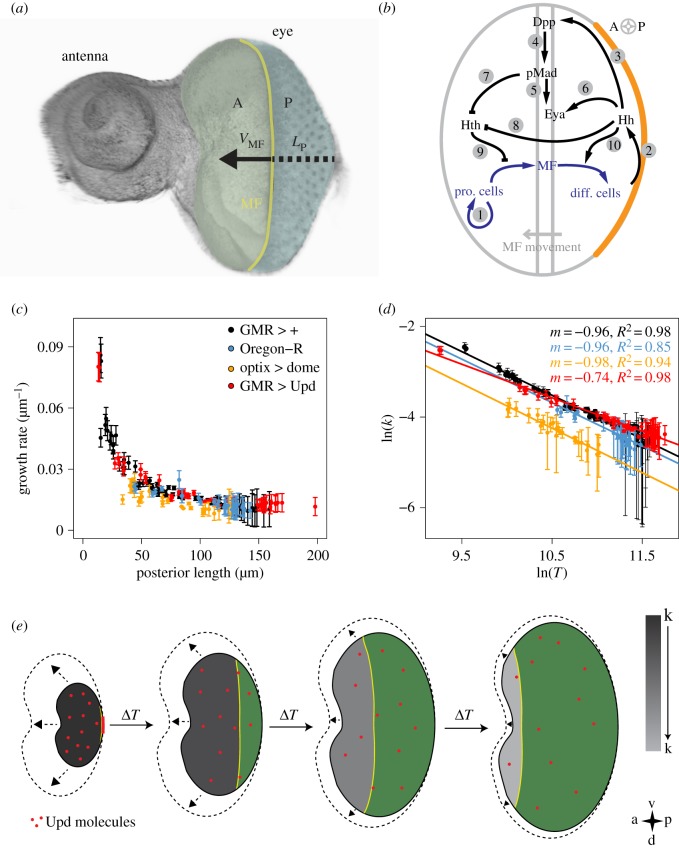
Growth control in the *Drosophila* eye imaginal disc. (*a*) Image of the eye-antenna imaginal disc and schematic illustration of the developmental process. The tissue parts forming the future eye and antenna are labelled. The morphogenetic furrow (MF, yellow) is initiated at the posterior margin. As development progresses, the MF sweeps over the tissue in an anterior-to-posterior direction. A, anterior area; P, posterior area; *V*_MF_, speed of the MF; *L*_p_, posterior length. (*b*) The regulatory network controlling the advancement of the MF during eye disc development. In front of the MF, progenitor cells proliferate (arrow (A)1), while behind the MF cells differentiate and eventually form the ommatidia. Hh is expressed in the posterior margin (marked in orange), from where it diffuses into the eye disc (A2), and initiates expression of dpp in the MF (A3). Dpp signals by phosphorylation of Mad to pMad (A4). pMad (A5) and Hh (A6) enhance the expression of eya. Both pMad-mediated Dpp signalling (A7) and Hh signalling (A8) repress the expression of hth. Initially, Hth is present throughout the disc. As the Hth levels decline, progenitor cells can transit into MF cells (A9). Hh supports the differentiation process by inducing the differentiation of MF cells which, in turn, initiate Hh expression (A10). Thus, Hh and Dpp/pMad together drive the progression of the MF. (*c*) The growth rate *k* in the tissue anterior of the MF declines continuously in eye disc with different genotypes as indicated. The posterior length *L*_P_ is linearly related to developmental time [30]. (*d*) Consistent with growth control by dilution, the growth rate *k* declines inversely proportional to the total eye disc area *T* in control eye discs (black and blue). A reduction in the Upd concentration by ectopic expression of a soluble form of the Upd receptor Dome results in a lower maximal growth rate, but the growth rate *k* still declines inversely proportional to the total eye disc area *T* (yellow). Ectopic expression of *upd* behind the MF counteracts Upd dilution and results in a slower decline in the growth rate (red). (*e*) The dilution-based growth control mechanism. In the early stages of eye disc development, Upd molecules (red points) are produced at the posterior margin (red line) and spread over the small eye disc domain by diffusion. Upd production ceases at the onset of MF movement. As a result of the increase in the total eye disc area over time, the Upd concentration decreases by dilution. The growth rate, *k*, in the part anterior to the MF is directly proportional to the concentration of Upd (visualized from dark to light grey) and therefore declines inversely proportionally to the change in the total eye disc area. As a result, the area increase within a time interval Δ*T* is less, allowing the MF to catch up and terminate growth. Anterior is to the left, and posterior to the right. Green, posterior area; yellow, MF; dashed lines, growth within the next time step. Picture and legend in (*b*) were adapted and reproduced with permission from Fried *et al.* [85] (Copyright © 2016 Public Library of Science). The pictures in (*c–e*) and the legend for (*e*) were adapted from Vollmer *et al.* [86]. Reprinted with permission from The Company of Biologists (UK).

Cell differentiation poses another possibility to generate a declining growth rate over time. An organ system where the effects of cell differentiation can be studied particularly well is the *Drosophila* eye disc (figure 3*a*). Growth and proliferation are mainly restricted to the tissue anterior to the morphogenetic furrow (MF) [87]. The regulatory interactions between Hedgehog (Hh), which is expressed only behind the MF, Dpp, which is expressed in the MF activated by Hh, and Homothorax (Hth), which is expressed only in front of the MF, result in a travelling wave that propels the MF from the posterior to the anterior side of the eye disc (figure 3*b*) [85,88,89]. Once the MF reaches the anterior-most side of the eye disc, growth terminates. The movement of the MF alone could thus slow down and ultimately terminate growth. However, the determination of the growth rate in the anterior part of the eye disc revealed that the growth rate declines continuously (figure 3*c*) [90]. Growth termination is thus not achieved by cell differentiation alone.

### 2.9. Growth termination by dilution of a cytokine

Intriguingly, the growth rate declines inversely proportional to the total eye disc area (figure 3*d*) [90]. Growth control in the eye disc could therefore be achieved by the dilution of a cytokine (figure 3*e*). Indeed, the cytokine Unpaired (Upd) is expressed only before the initiation of the MF [91], such that the maximal concentration is set before the start of the differentiation process. Also, Upd is sufficiently long-lived (approx. 60 h) that its concentration is mainly reduced by growth-dependent dilution rather than by protein turn-over [86]. Biochemical studies further show that the intracellular JAK/STAT pathway responds about linearly to the Upd levels [92,93]. The comparably high diffusion coefficient further ensures that the Upd concentration remains rather uniform in spite of spatial inhomogeneities in growth [86]. Consistent with a dilution mechanism, mutants with lower Upd levels have smaller eyes, but the area growth rate still declines inversely proportional to the total eye disc area (figure 3*d*, blue and yellow lines) [86]. Mutants that express Upd ectopically behind the MF have much bigger eyes, and the growth rate no longer declines proportionally to area growth (figure 3*d*, red line) [86]. In summary, the observations in the *Drosophila* eye disc are consistent with growth control by dilution of the cytokine Upd. The mechanism is, however, specific to the eye disc as the growth data from the *Drosophila* wing disc cannot be explained with a dilution mechanism [94]. The dilution mechanism thus does not represent a general mechanism for growth termination, and other mechanisms must operate in other organs and appendages.

### 2.10. The intercalation model

**Figure 4.**
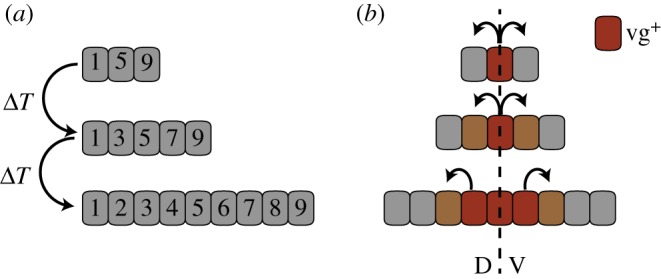
Intercalation models. (*a*) The intercalation model. According to the intercalation model, cells possess a positional value that is assigned at their ‘birth’ (here indicated by a number). Proliferation is assumed to be proportional to the difference in the positional value between neighbouring cells. ‘Newborn’ cells intercalate between the cells and assume an intermediate positional value. Proliferation stops as the difference in the positional values of neigbouring cells becomes too small. (*b*) The feed-forward model proposes that cells are recruited to a wing pouch fate starting from the dorso (D)–ventral (V) axis. Cells that have been recruited to a wing pouch fate start expressing *vestigial* (vg^+^, red) and are then capable of inducing this fate in neighbouring cells. At the same time, this mechanism generates a biochemical differential that is translated into increased proliferation.

According to the intercalation model, cells possess some kind of positional value that is assigned to each cell at its ‘birth’ and remains invariant (figure 4*a*) [95–98]. The rate of proliferation/growth depends on the difference in positional value between neighbouring cells. Daughter cells intercalate between the original cells, and assume an intermediate positional value. Growth terminates once the difference in the positional value between neighbouring cells is smaller than some threshold [98].

The mechanism was originally proposed to explain the regeneration of newt limbs [36], but has been applied to a wide range of regenerating tissues, including the *Drosophila* imaginal discs [99,100]. According to the intercalation model, the blastema (i.e. the stem cell population that regenerates the missing tissue) that initially covers the amputated limb defines ‘distal’, while the proximal stump preserves its positional identity. During regenerative regrowth, the missing positional values would be intercalated progressively. Indeed, the kinetics of newt limb regeneration depend on the size of the amputated structure and decline as regrowth progresses, such that regeneration of a proximal and distal amputate take similar times [101]. To permit a direct experimental confirmation of an intercalation mechanism in organ/appendage size control, the molecular details of the postulated cell identity gradient need to be defined. Here, it will be important to understand how a cell identity gradient can be established and read reliably across a large developmental field, as well as the cross-talk with extrinsic size control (i.e. how the positional identity would scale when embryonic structures grow to different finite sizes, for instance because of differences in available nutrients).

### 2.11. The feed-forward model

Like the intercalation model, the feed-forward model is based on cell–cell interactions, but also depends on Wingless (Wg) as a morphogen. Proposed by Zecca & Struhl [102–104], the idea behind this model is that cells are recruited to a wing fate starting from the dorsoventral (DV) boundary (figure 4*b*). After the initial trigger of the wing-pouch-selector gene *vestigial* (*vg*) and of *wingless* (*wg*, a Wnt morphogen) by the DV signalling centre, the growth of the wing pouch region would proceed through a double process: recruitment of non-pouch cells as *vg-*expressing pouch cells and the induction of proliferation in these non-pouch cells. The mechanism for the developmental ‘expansion’ of the wing requires the generation of a sharp differential in the expression of Fat and Ds at the edge of the expanding domain, which results in the repression of the Hippo pathway and the activation of Yki. The result of this activation would be twofold: an increase of proliferation and the initiation of *vg* expression, thus allowing the feed-forward induction of *vg* and expansion of the wing*.* Although not directly addressed by these authors, their model could, in principle, explain growth termination based on the dependence of the feed-forward expansion on *wg. wg* is detected as a gradient with a maximum at the pouch's centre (along the DV) and tapering off towards its periphery. Therefore, if there were a quantitative dependence on Wg, the expansion and the associated proliferation would decay until Wg's concentration would fall under a threshold (i.e. far from the disc's centre). This model has, however, recently been challenged by the finding that a membrane-tethered form of Wg can replace the endogenous, diffusible protein resulting in normally patterned and sized wings [105]. However, one could imagine that a similar result would arise if, as time progresses, *wg* expression becomes progressively more restricted to the disc centre, something that is likely the case [105–107]. Still, this model neither explains how proliferation is maintained throughout the pouch in the wake of the expanding front, nor the deceleration in proliferation rate with developmental time.

### 2.12. Models based on tissue mechanics

Finally, mechanical constraints have been proposed to limit growth. Two similar, but still distinct, mechanical models have been put forward. In 2005, Shraiman [108] proposed that a clone of cells which is growing faster than its surrounding is experiencing mechanical stress. Assuming a putative ‘integral-feedback’ in which this stress reduces the growth rate of the cells, Shraiman [108] suggested that this would result in a uniform growth rate throughout the disc. Later, the model was extended to account also for growth termination [109]. In this model, growth stops as cells at the lateral parts stop proliferating when they fall below a Dpp threshold. Cell cycle arrest in the lateral parts of the tissue then leads to an increase of mechanical stress in the centre of the disc. Based on the putative feedback, that stress reduces the growth rate, this ultimately leads to growth termination [109]. Importantly, this model requires that the Dpp gradient does not scale with tissue size [109], in conflict with the findings by Wartlick *et al.* [30].

**Figure 5.**
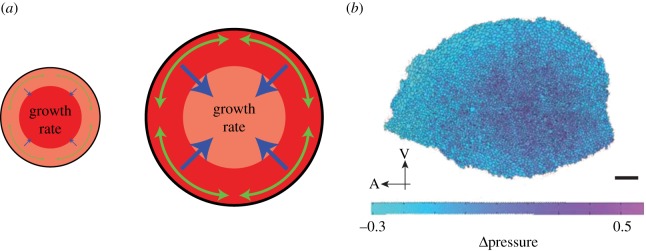
Growth control by mechanical feedbacks. (*a*) Illustration of the model developed in [110]. The *Drosophila* wing disc pouch is shown as an idealized circle here. At the beginning, most growth occurs in the centre of the disc (left, dark red indicating a higher growth rate). As the discs grows, lateral cells get stretched (green), inducing growth in this region. This is, however, insufficient to compensate for the stretching. Therefore, the centre of the disc gets compressed (blue), reducing the growth rate. Growth terminates once cells cannot anymore overcome the inhibitory effect by the compression. (*b*) Distribution of hydrostratic pressure differences, Δ*P*, in a wing disc, as inferred from the cell geometries: the hydrostatic pressure difference is higher in the centre of the wing disc than in the periphery; the values were normalized such that average pressure difference in the tissue is zero (colour bar). Adapted from Ishihara & Sugimura [112]. Reprinted with permission from Elsevier. Scale bar, 20 µm.

The models developed by Aegerter-Wilmsen *et al.* [110,111], on the other hand, require the exact contrary, namely the scaling of the gradient. Similar to the models by Hufnagel, Shraiman and co-workers [108,109], they assume that compression leads to inhibition of growth. Furthermore, they assume, however, that stretching above a certain threshold induces growth [110]. Finally, they require another morphogen gradient perpendicular to Dpp, which also scales with tissue size. This model thus includes the major genetic data used to build the feed-forward model (see above). Based on these assumptions, they built a model in which Dpp induces growth in the medial part of the disc. This growth leads to a tangential stretching of the lateral parts. Even though this stretching induces growth in those parts, it cannot completely compensate the compression (figure 5*a*). As a net result, the compression of the central part of the disc increases, eventually resulting in growth termination [110,111]. Indeed, differential proliferation rates between disc's centre and periphery as well as uneven stress and pressure distributions were later experimentally observed in the wing disc, with maximum compression and highest hydrostatic pressure in the centre (figure 5*b*) [113–115]. One key observation that these models fail to explain is the autonomous growth of the anterior and posterior compartments of the tissue. As described above, if either compartment has a *Minute* mutation, the two compartments grow with different speeds, while the final disc size is comparable to that of wild-type discs [45]. This cannot easily be explained by the proposed models. It is also unknown to what extent the actual buckling of the disc has to be taken into account, or whether the observed distribution of mechanical stress can actually lead to this buckling.

Finally, it is an open question how cells sense the mechanical status of their environment. Recent work implicates the Hippo pathway [116,117] as an important component of the mechanism required for mechanical stress feedback. In *Drosophila*, the status of the acting cytoskeleton (which controls cell shape and, together with myosin, regulates rheological properties of the cell cortex) is linked to the Hippo pathway, a growth regulator [118,119]. A role of the Hippo pathway in mechanotransduction has been proposed also in vertebrate cells [120,121]. Therefore, there is a potential signalling/biochemical link between mechanical forces and growth control. More recently, Parker & Struhl [122] showed that, in the *Drosophila* wing disc, Yki can be secluded in the nucleus, thus made unable to access its transcriptional targets, unless the TOR pathway (which links nutrition to growth) is active. Therefore, the activity of the Hippo pathway—and specifically through the regulation of Yki—seems to be at the crossroads of mechanical forces, nutrition and growth, and thus is becoming centre-stage in the study of the regulation of growth termination (i.e. size). However, recent work by Ma *et al.* [123], in which mechanical tension on the wing epithelium was modified by changing the composition of its basal membrane, challenges the idea that mechanical feedback has a main role in growth control.

## 3. Conclusion and outlook

The ability to coordinate growth between organs and to terminate growth at a set final organ size was a key step in the evolution of complex organisms. Within this review, we have presented the models and ideas proposed to explain growth termination in the development of the *Drosophila* wing and eye disc. Even though each of these models was treated separately here, it is possible that a combination of several mechanisms contribute to growth termination control.

Intriguingly, whichever the final mechanism(s) of growth termination may be, it should allow the readily adaption to changes in environmental conditions and overall organism size, while being extraordinarily robust to a range of perturbations, including those in cell size and numbers. One general rule, found across the animal kingdom, is that the rate of growth declines as development progresses [124]. Future quests for the mechanism of growth termination will have to take the wide range of experimental observations into account and ideally explain the observed growth dynamics across tissues, species and ecological/environmental and experimental conditions.

## Data accessibility

This article has no additional data.
